# Quantification of Intracellular Thiols by HPLC-Fluorescence Detection

**DOI:** 10.3390/molecules26082365

**Published:** 2021-04-19

**Authors:** Hiroki Yamamoto, Takuya Fujiwara, Takashi Funatsu, Makoto Tsunoda

**Affiliations:** Graduate School of Pharmaceutical Sciences, University of Tokyo, Tokyo 1130033, Japan; drinkingrabbit1672@gmail.com (H.Y.); taku1729@gmail.com (T.F.); funatsu@mol.f.u-tokyo.ac.jp (T.F.)

**Keywords:** cysteine, glutathione, derivatization, oxidative stress

## Abstract

Biothiols, such as cysteine and glutathione, play important roles in various intracellular reactions represented by the redox equilibrium against oxidative stress. In this study, a method for intracellular thiol quantification using HPLC-fluorescence detection was developed. Thiols were derivatized with a thiol-specific fluorescence derivatization reagent, viz. ammonium 7-fluoro-2,1,3-benzoxadiazole-4-sulfonate (SBD-F), followed by reversed-phase separation on an InertSustain AQ-C18 column. Six different SBD-thiols (homocysteine, cysteine, cysteinylglycine, γ-glutamylcysteine, glutathione, and *N*-acetylcysteine as an internal standard) were separated within 30 min using a citric buffer (pH 3.0)/MeOH mobile phase. The calibration curves of all the SBD-thiols had strong linearity (R^2^ > 0.999). Using this developed method, the thiol concentrations of human chronic myelogenous leukemia K562 cell samples were found to be 5.5–153 pmol/1 × 10^6^ cells. The time-dependent effect of a thiol scavenger, viz. *N*-ethyl maleimide, on intracellular thiol concentrations was also quantified. This method is useful for elucidating the role of intracellular sulfur metabolism.

## 1. Introduction

**Figure 1:**
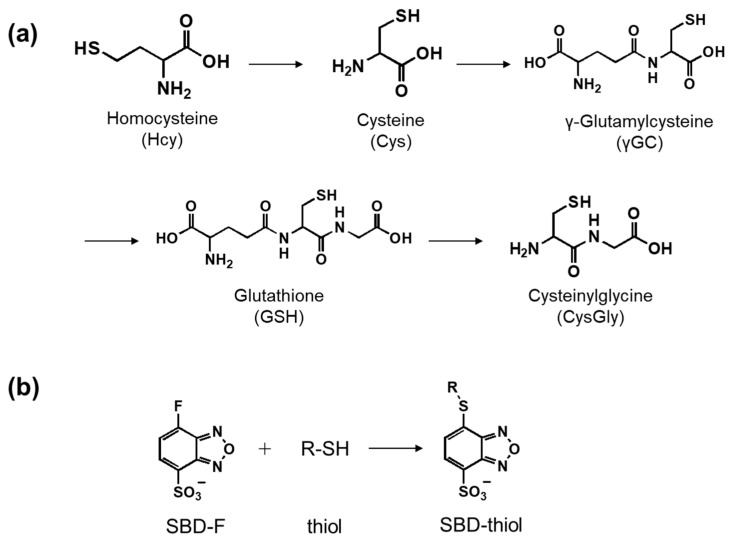
(**a**) Metabolic pathway of thiols and (**b**) derivatization reaction of SBD-F with thiols.

Biological thiols, such as cysteine (Cys) and glutathione (GSH), are ubiquitous in biological tissues and fluids (Figure 1a). Thiols play important roles in various intracellular reactions against oxidative stress, such as in redox equilibria. The alteration of intracellular thiol concentrations has been correlated with health conditions, such as liver disease, cancer, and cardiovascular disease [1,2,3]. For example, lung cancer causes a decrease in the intracellular concentrations of Cys and cysteinylglycine (CysGly), while prostate cancer leads to increased levels of Cys [4,5]. Thus, the detection of intracellular thiol imbalance is important for elucidating the role of thiols in diseases.

The main analytical techniques that are available for characterizing intracellular thiols include the use of optical probes and separation-based methods. Optical probes, mostly fluorescent ones, enable the selective detection of thiols with high sensitivity [6,7,8]. Several types of probes have been developed for bioimaging applications; however, they do not allow for accurate quantification and are mostly non-selective among thiols, and can be used to either detect the total thiol amount or selectively detect one particular thiol [9]. On the other hand, separation-based analytical methods can achieve accurate and simultaneous quantification of several different thiols.

Currently available separation-based analytical methods for thiols include HPLC-fluorescence detection, LC-mass spectrometry, and capillary electrophoresis [10,11,12,13]. We have previously developed analytical methods for thiol detection based on fluorescence derivatization using ammonium 7-fluoro-2,1,3-benzoxadiazole-4-sulfonate (SBD-F) (Figure 1b) and hydrophilic interaction chromatography (HILIC) [14,15,16,17]. Although eight types of thiols could be separated within 10 min with high sensitivity, these methods were difficult to apply in routine analyses, owing to the low stability of the HILIC column, the long time required for column equilibrium, and limitations as regards the injection sample volume [18,19,20]. Therefore, in this study, we developed a more stable analytical method for quantifying intracellular thiols using an ODS column.

## 2. Results and Discussion

### 2.1. Investigation of SBD-Thiols Separation

First, the separation of SBD-thiols was performed on an Inertsil ODS-4V column. However, sufficient separation could not be obtained under any of the mobile phase conditions examined. Particularly, three SBD-thiols, viz. Hcy, CysGly, and γGC, were not well separated owing to the insufficient retention of these SBD-thiols on the ODS-4V column.

**Figure 2:**
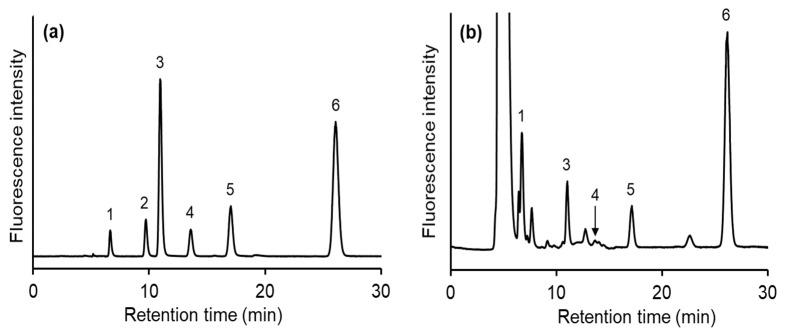
Chromatograms of (**a**) the SBD-thiol standards and (**b**) the K562 cell sample. Mobile phase: 100 mM citric buffer (pH 3.0)/MeOH (95/5, *v*/*v*). Column: InertSustain AQ-C18 (250 × 3.0 mm I.D., 5 μm). Peaks: 1; SBD-Cys, 2; SBD-Hcy, 3; SBD-CysGly, 4; SBD-γGC, 5; SBD-GSH, 6; SBD-NAC.

Therefore, the ODS-4V column was replaced by an InertSustain AQ-C18 column, which has a stronger retention level. As expected, using the same mobile phase as for the ODS-4V column, all the SBD-thiols showed better retention on the AQ-C18 column. When a mobile phase of 50 mM sodium acetate buffer (pH 4.0)/MeOH (97.5/2.5, *v*/*v*) was used, most of the SBD-thiols were separated; however, SBD-Hcy and SBD-γGC were not successfully separated. When the MeOH content in the mobile phase was decreased, sufficient separation was still not achieved. Thus, for the separation of SBD-Hcy and SBD-γGC, their logD values, which are predicted values of the lipophilicity of ionizable compounds, were calculated using SPARC (ARChem, Danielsville, GA, USA). These values are pH-dependent and indicate different solubilities in organic solvent/water. A high logD value indicates that the compound has low polarity. When the pH was changed from 4.0 to 3.0, the logD value of SBD-Hcy decreased slightly, while that of SBD-γGC increased, suggesting that the hydrophobicity of SBD-γGC decreased compared to that of SBD-Hcy. Since the acetate buffer has minimal buffering ability at pH 3.0, citrate buffer was used instead. The optimal MeOH content in the mobile phase for fast separation was found to be 5%. Accordingly, six different SBD-thiols were separated within 30 min using a mobile phase of 100 mM citric buffer (pH 3.0)/MeOH (95/5, *v*/*v*) (Figure 2a).

### 2.2. Application to K562 Cell Samples

The method developed in this study was applied to human chronic myelogenous leukemia K562 cell samples. Although all the peaks were observed under optimized conditions, the peak corresponding to SBD-GSH was beyond the detection range because of a difference of one order of magnitude between intracellular concentrations of GSH and the other thiols. This problem could be solved by diluting the injection samples; however, when diluted, some SBD-thiols could not be detected. Therefore, the sensitivity of the fluorescence detector was changed by time-programming according to thiol concentrations: SBD-Cys, SBD-γGC, and SBD-CysGly concentrations were measured at a gain of 1000, while the SBD-GSH concentration was measured at a gain of 10. A typical chromatogram of a cell sample is presented in Figure 2b. SBD-Hcy was not detected on this chromatogram. The intracellular concentrations of Cys, CysGly, γGC, and GSH were 32.1 ± 1.5, 40.1 ± 2.3, 5.5 ± 0.4, and 153 ± 3 pmol/1 × 10^6^ cells, respectively (*n* = 4). Among all the thiols examined, GSH was the most abundant in cells, which is in good agreement with the results of previous studies [21,22]. However, the cells contained significant concentrations of other thiols. Interpreting the results of the thiol-selective imaging probe was important because some previous studies have shown that intracellular GSH concentrations are equal to total thiol concentrations [9].

### 2.3. Method Validation

Table 1 lists the validation data for the developed method. Good linearity was obtained, with correlation coefficients exceeding 0.999. The thiol sensitivity was lower than that obtained with HILIC methods [14,15,16,17] and was similar to those obtained in other studies [23,24], except for GSH. This was because the sensitivity of the fluorescence detector for GSH was lowered. The intra- and inter-day precisions were 2.2–8.4% and 1.8–13.7%, respectively, while their accuracies were 91–107% and 94–124%, respectively. These validation data showed that the developed method was suitable for the routine analysis of thiols in the cell samples.

### 2.4. Investigation of Intracellular Thiols by N-Ethyl Maleimide (NEM) Addition

**Figure 3:**
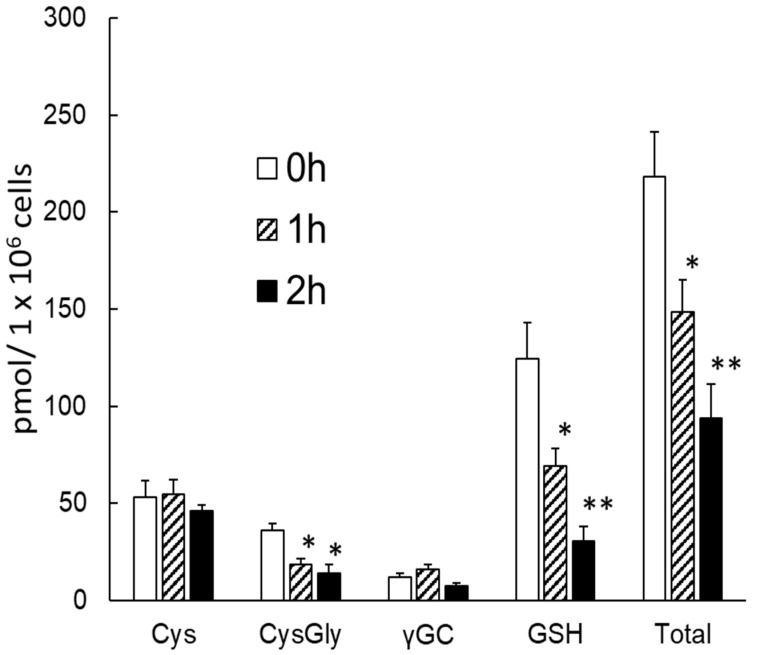
Changes of thiol concentrations after NEM addition (*n* = 6, mean ± SEM). Differences in multiple groups were assessed by the non-paired Student’s *t*-test with the Bonferroni correction. * *p* < 0.05, ** *p* < 0.01 vs. 0 h.

To investigate whether the developed method could be used to detect changes in intracellular thiols, NEM was used to modulate intracellular thiol concentrations. Although NEM has been used in many studies by researchers, most of them reported a decrease in the total or specific thiol concentration after NEM addition. Hence, it is interesting to examine concentration changes in each thiol, specifically Cys, CysGly, γGC, and GSH, after NEM addition. Intracellular thiol concentrations at 1 h and 2 h after NEM addition were quantified using the developed method, and the changes in Cys, CysGly, γGC, GSH, and total thiol concentrations are shown in Figure 3. GSH and total thiol concentrations significantly decreased in a time-dependent manner, with less than half the initial amount remaining after 2 h, which is consistent with the results of previous studies [25,26]. Among the other thiols, the CysGly concentration showed a downward trend, similar to that of GSH. This is a reasonable result because CysGly is produced by metabolism of GSH (Figure 1). Changes in Cys and γGC concentrations were different from those in GSH and CysGly concentrations, but they did not decrease significantly after 2 h. This was unexpected, because NEM is known as a thiol scavenger; further studies are necessary to elucidate this phenomenon. However, it can be speculated that the syntheses of Cys and γGC might be enhanced to compensate for the decrease in their concentrations after NEM addition.

## 3. Materials and Methods

### 3.1. Chemicals

Cysteine (Cys), homocysteine (Hcy), cysteinylglycine (CysGly), and γ-glutamylcysteine (γGC) were from Sigma-Aldrich (St. Louis, MO, USA). Glutathione (GSH), *N*-acetylcysteine (NAC), sodium acetate, citric acid, potassium dihydrogen citrate, and tris(2-carboxyethyl) phosphine (TCEP) were obtained from FUJIFILM Wako Pure Chemical (Osaka, Japan). Ammonium 7-fluoro-2,1,3-benzoxadiazole-4-sulfonate (SBD-F) was obtained from Dojindo Laboratories (Kumamoto, Japan). MeOH (HPLC grade) was from Merck KGaA (Darmstadt, Germany). A Milli-Q system (Merck) was used for water purification.

### 3.2. Cell Culture and Treatments

K562 cells (obtained from American Type Culture Collection, ATCC, Rockville, MD, USA) were cultured in Roswell Park Memorial Institute 1640 medium (RPMI-1640) containing 10% FBS, 100 IU/mL penicillin, and 100 mg/mL streptomycin. Cells were washed with phosphate-buffered saline (PBS), and were treated with 80% methanol. After removing insoluble particles by centrifugation, supernatants were collected and dried under vacuum. Dried cell samples were dissolved in 75 μL of water (cell solutions). As for the *N*-ethyl maleimide (NEM) addition experiment, 1 mM NEM was added to the medium, and K562 cells were cultured for 0, 1, and 2 h. Cultured cells were treated as above.

### 3.3. Derivatization Conditions

TCEP solution (120 mg/mL, 5 μL) was added to 75 μL of thiol aqueous solution in a sealed tube. After 30 min, it was centrifuged at 15,000 g for 10 min. To the supernatant (50 μL), 175 μL of SBD-F solution (0.86 mg/L) and 25 μL of NAC solution (60 μM) were added, and heated at 60 °C for 60 min. Twenty-five microliters of 1 M HCl was added to the solution, and 25 μL was injected into HPLC system.

### 3.4. HPLC Conditions

HPLC analysis was performed on Jasco HPLC systems (Tokyo, Japan). SBD-thiols were separated on Inertsil ODS-4V (250 × 3.0 mm I.D., 5 μm) or InertSustain AQ-C18 (250 × 3.0 mm I.D., 5 μm) (GL Sciences, Tokyo, Japan). 100 mM citric buffer (pH 3.0)/MeOH was used as mobile phase and flow rate was 0.3 mL/min. The column temperature was 40 °C. Fluorescence detection was performed with excitation and emission wavelengths of 375 and 510 nm, respectively. Gain of the fluorescence detector was programmed as follows, 0–15.5 min: 1000, 15.5–21 min: 10, and 21–30 min: 100.

### 3.5. Validation

Calibration curves were calculated by the ratio of peak area of each SBD-thiol to that of SBD-NAC (internal standard). Concentration ranges were 50–5000 nM, 5–500 nM, 1–100 nM, and 100–10,000 nM for SBD-Cys, SBD-CysGly, SBD-γGC, and SBD-GSH, respectively. Intra- and inter-day precisions and accuracies were determined as its relative standard deviation (RSD) by measuring cell sample five times within the same day and on four consecutive days, respectively.

## 4. Conclusions

In this study, we developed an analytical method for detecting and quantifying intracellular thiols that can be applied to K562 cell samples. Thiols were first reduced by TCEP and then fluorescently derivatized by SBD-F. Six different SBD-thiols were successfully separated within 30 min on an ODS column, which can perform more stable analyses than HILIC columns [14,15,16,17]. By adjusting the sensitivity of the fluorescence detector, Cys, CysGly, γGC, and GSH in K562 cells could be quantified simultaneously. The results of this study can be considered to shed light on intracellular sulfur metabolism via thiol quantification.

## Figures and Tables

**Table 1:** Validation data of the developed method.

Thiol	Linearity	Intra-Day		Inter-Day	
	(nM, R^2^ > 0.999)	RSD (%)	Accuracy (%)	RSD (%)	Accuracy (%)
Cys	50–5000	4.3	98	13.7	105
CysGly	5–500	4.0	91	5.8	95
γGC	1–100	8.4	107	9.2	124
GSH	100–10,000	2.2	95	1.8	94
